# Work-related musculoskeletal problems related to laboratory training in university medical science students: a cross sectional survey

**DOI:** 10.1186/s12889-018-6125-y

**Published:** 2018-10-29

**Authors:** Stefania Penkala, Hannan El-Debal, Kristy Coxon

**Affiliations:** 1 School of Science and Health, Western Sydney University, Locked Bag 1797, Penrith, Sydney, NSW 2751 Australia; 2 Translational Health Research Institute, Western Sydney University, Locked bag 1797, Penrith, Sydney, NSW 2751 Australia

**Keywords:** Musculoskeletal diseases epidemiology, Occupational health, Ergonomics, Laboratory activities

## Abstract

**Background:**

Work-related musculoskeletal problems impact everyday function, working ability, and quality of life. Unaddressed musculoskeletal problems can lead to major injury and loss of function, contributing to participation restrictions, economic loss and the increasing burden of disease worldwide. Medical science laboratory technicians are not immune with reported work-related musculoskeletal problems between 40 and 80%. Similar data is not available for medical science students, who may be the most vulnerable at the beginning of their careers. This study investigated the prevalence, common sites, impact and potential solutions for work-related musculoskeletal problems in medical science students during their university laboratory training.

**Methods:**

A Standardised Nordic Musculoskeletal Questionnaire was administered to medical science students at a local university in Sydney, Australia, to evaluate the prevalence, site and impact of work-related musculoskeletal problems. Problems were defined as an ache, pain, discomfort or numbness in body regions within 12 months and last 7 days in this period. The questionnaire was administered between April and June 2017.

**Results:**

The response rate was 38.2% (*n* = 110/288). Over a third (*n* = 38/110) reported a laboratory related musculoskeletal problem in the last 12 months and just over a fifth (*n* = 24/110) within 7 days. The lower back (30% and 17%), neck (24% and 10%) and upper back (21% and 10%) were the most common sites of problems reported within a 12 month and 7 day period respectively. Problems reported in the lower back, neck and upper back prevented daily activities in the majority of cases (between 63 to 83%) with many seeking physician or health professional assistance (between 13 to 83%). Solutions suggested by respondents included better seating designs, rest periods and education about correct working posture.

**Conclusions:**

Some medical science students during their laboratory training are already experiencing high levels of musculoskeletal problems, even before they enter the workforce. While the response rate was low affecting generalizability, the extent of problems limiting activity and needs to seek assistance of those reporting problems is of concern. Strategies are suggested to address ergonomic and postural training, as part of university curriculums, including the identification of problems for early intervention to facilitate sustainable workforces.

## Background

Work-related musculoskeletal disorders are the most prevalent health issue faced by the working population [1], and are associated with more absenteeism or disability than any other diseases [2]. An estimated 6.1 million Australians (28% of the total population) in 2010–11 [3], accounted for 60% of serious workplace compensation claims in a five year period [1]. Disorders not only affect an individual’s ability to work and function in daily life, but also exert an economic impact on the workplace, health system and the community [4].

The cost of musculoskeletal conditions and arthritis in Australia alone, in 2012, were estimated to be $55.1 billion [1], and overall workers compensation and associated private costs were $250 billion in the United States [5]. While health and medical workers are part of the team to manage and prevent workplace injury, they are also overrepresented in workplace injury statistics [4]. Burnout, high staff turnover rates, and poor job satisfaction have also been attributed to health care workforce shortages and musculoskeletal disorders [4, 6, 7]. Workplace injury research in the health workforce has mostly investigated nursing personnel [8]. However, other healthcare workers are also at risk [9–12], including medical laboratory technicians [13].

Workplace laboratory tasks often require repetitive, fine task precision, static, sustained and awkward postures which increase musculoskeletal injury risk [13]. The cumulative risk includes micro trauma from routine tasks such as pipetting, microscopy, micromanipulation, and working with biosafety cabinets or cryostats [13]. Time constraints and accuracy demands inherent in laboratory tasks, coupled with productivity pressures heighten the work-related musculoskeletal risk [13]. Female gender and increased working hours can further contribute to the prevalence of musculoskeletal problems [14–16]. Despite attention to ergonomic issues and improvements in equipment and workplace design, laboratory technicians still report a high prevalence of workplace injury [13].

The prevalence of laboratory work-related musculoskeletal problems have been reported to be between 72% [16] and 80% [17] in Iran and India, in studies with sample sizes between 49 and 156, using the validated Standardised Nordic Musculoskeletal Questionnaire (SNMQ) [18]. The lower back (31 to 43%) and neck (18 to 33%) are common sites of laboratory work-related musculoskeletal problems [16, 17]. Workplace musculoskeletal problems within the SNMQ are defined as an ache, pain, discomfort or numbness.

Prevalence of laboratory work-related musculoskeletal problems are relatively consistent in studies using different questionnaires, reporting the neck and lower back as the more prominent sites of problems or injuries overall (9 to 60%) in studies from America [19, 20], Ethiopia [15], Iran [21], Sweden [22] and Switzerland [14]. The shoulders (58 to 60%), upper back (25 to 57%) and hands/wrists (28 to 57%) have also been reported to be prominent sites of musculoskeletal problems or injuries as well, particularly with high involvement in pipetting and microscope activities [14, 19, 20, 22]. Problems in knees, ankles and feet (10 to 20%) can also be prevalent with prolonged standing and moving across the workplace for different equipment needs [15, 17, 22]. Variations in reported prevalence of laboratory work-related musculoskeletal problems, in part can be explained by the use of different survey tools and data collection timelines (i.e. one month versus 12 months), inconsistency in terminology (i.e. injury versus symptoms or problems), and different working environments, job roles and stressors.

While there is a continuing evidence base about work-related musculoskeletal problems amongst laboratory professionals, there is a lack of data specific to the Australian context. Furthermore, the prevalence of laboratory work-related musculoskeletal problems within medical science students, during their training, who may be at most risk early in their careers [12, 14] is unknown. As laboratory environments, even in training, require adaptation to fixed working positions and postures for extended periods, accompanied with repetitive activities, students new to these activities are likely to be at an increased risk [14]. Almost half of reported workplace musculoskeletal injury in the health workforce is reported in the first five years of practice [12]. Appropriate ergonomic training, including musculoskeletal exercises, developed in a participative manner and introduced into the workplace and university curriculums is recommended [10, 20, 23].

The aim of this study was to investigate the prevalence, common sites, impact and potential solutions of laboratory work-related musculoskeletal problems amongst medical science students in training, occurring over both a 12 month and an immediate 7 day recall period. Understanding the risk and prevalence among students is an important first step to inform current teaching methods and intervention strategies to limit future musculoskeletal problems in laboratory students and technicians.

## Methods

### Design and participants

A retrospective cross-sectional survey investigating the prevalence of laboratory work-related musculoskeletal problems was administered to medical science students enrolled at a large multi-campus university in Sydney, Australia. Medical science students enrolled in the three year program were invited via email (with one follow up email) to participate in the online survey or paper-based copy. One class list from each year was used to identify current students, and no incentives were available for participation. Musculoskeletal data were collected using the Standardised Nordic Musculoskeletal Questionnaire (SNMQ) which is a reliable and valid tool with test-retest and validity agreement values between 77 and 100% [18]. Ethics approval was obtained from the Western Sydney University Human Research Ethics Committee (approval number: H9563).

### Standardised Nordic musculoskeletal questionnaire (SNMQ)

The SNMQ includes questions about musculoskeletal problems experienced, defined as an ‘ache, pain, discomfort or numbness’ in the last 12 months and more immediate 7 day recall periods, and their impact on participation in daily activities and need to seek physician or health professional assistance. The 7 day recall component of the survey was only completed if a problem occurred in the 12 month period. The SNMQ was modified to capture reported musculoskeletal problems that occurred only in relation to student laboratory work-related training activities. Sites for potential laboratory work-related musculoskeletal problems were identified as the neck, shoulders, upper back, elbows, wrist/hand, lower back, hip/thigh, knees, and ankles/feet. The survey was also modified to include demographic information and specific information relevant to medical student laboratory training. Questions included gender, age, year of study, ergonomic positions, hours of laboratory practice, percentage of time participating in specific laboratory activities (i.e. pipetting), perceived postural awareness and open ended questions about their concerns.

### Data analysis

All data were entered onto IBM SPSS version 23 Windows software for statistical analysis. Descriptive statistics were used to describe the sample. Means and standard deviations (SD) were used to describe the data when normally distributed and medians and interquartile ranges (IQR) when not. Categorical data were described in percentages, to represent proportions of the sample. Missing data were less than 5% and were excluded in the statistical analysis. Pearson chi square analyses were undertaken to determine any associations between categorical data including work-related musculoskeletal disorders, gender, work postures and ergonomic exercises. One way ANOVAs were used to identify any relationships between numerical and categorical data. A directed content analysis [24] was used to analyse written comments until unique themes were identified.

## Results

### Participation and sample description

A total of 110 of 288 medical science students across three university campuses completed the survey, representing a 38.2% response rate. The mean age of students was 21 (± 3.4) years, with the majority female (69.1%), and participating in 5.7 (±2.5) hours of laboratory activities per week (Table 1). The majority of laboratory time was spent on desk and pipetting activities (median 40 and 30% of time respectively). Non-adjustable stools were the most common seating used during laboratory activities (71.8%). Half of participants worked in a seated position for the majority of the time (Table 1). Thirty eight percent of medicine science students indicated they had poor posture, with 24% indicating they had a non-standard range of motion at their joints.

**Table 1:** Participant Characteristics

Characteristics	
Age, mean (SD)	21 (3.4)
Gender, *n*(%)	
Male	32 (29.1)
Female	76 (69.1)
Other	2 (1.8)
Year of Study, *n*(%)	
Year 1	30 (27.3)
Year 2	31 (28.2)
Year 3	47 (42.7)
Missing data	2 (1.8)
Weekly Laboratory Hours, mean (SD)	5.7 (2.5)
Percentage time in Laboratory Activity, median (IQR)	
Fume Cupboard	5 (2–10)
Pipetting	30 (20–40)
Desk Activities	40 (30–50)
Waiting for Samples (i.e. Centrifuging)	10 (5–20)
Other	0 (0–5)
Perceived Posture, *n*(%)	
Poor	42 (38.2)
Good	62 (56.4)
Excellent	6 (5.5)
Main Workplace Position, *n*(%)	
Seated	56 (50.9)
Standing	21 (19.1)
Equal Sitting and Standing	33 (30.0)
Main Type of Seating	
Adjustable Chair	31 (28.2)
Stool	79 (71.8)
Hand Dominance, *n*(%)	
Right	93 (84.6)
Left	12 (10.9)
Ambidextrous	5 (4.5)
Perceived Joint Mobility, *n*(%)	
Within Normal Limits	81 (73.6)
Hypermobile	20 (18.2)
Hypomobile	9 (8.2)
Performs Ergonomics Exercises, *n*(%)	
Yes	65 (59.1)
No	45 (40.9)

### Prevalence and sites of work-related musculoskeletal problems

Overall, 34.5% (*n* = 38/110) of the participants reported a laboratory work-related musculoskeletal problem in the last 12 months. In the more immediate time frame of 7 days, 21.8% (*n* = 24/110) of participants reported a laboratory related musculoskeletal problem, which accounted for 63.2% (n = 24/38) of the same students reporting a problem in 12 months. The mean number of concurrent sites of problems reported by students in the 12 months and 7 day recall periods were 3.4 (±1.6) and 2.8 (±1.6) respectively. The most common sites of reported musculoskeletal problems in the last 12 months were the lower back (27.3%), neck (23.6%), upper back (20.0%), and shoulders (15.5%; Table 2). The lower back (15.5%), neck (10.0%), upper back (13.6%) and shoulders (6.4%) were also the most common sites reported in the last 7 days (Table 2).

**Table 2:** Overall prevalence of laboratory work-related musculoskeletal problems within the last 12 months and 7 days (Total sample = 110)

Body area	Problems within last 12 months	Problems preventing daily activities	Problems requiring physician or health professional assistance	Problems within last 7 days	Problems preventing daily activities	Problems requiring physician or health professional assistance
	n (%)	n (%)	n (%)	n (%)	n (%)	n (%)
Neck	26(23.6)	21(19.1)	16(14.5)	11(10.0)	7(6.4)	2(1.8)
Shoulders	17(15.5)	10(9.1)	9(8.2)	7(6.4)	4(3.6)	2(1.8)
Upper back	22(20.0)	16(14.5)	14(12.7)	15(13.6)	10(9.1)	2(1.8)
Elbows	1(0.9)	1(0.9)	1(0.9)	1(0.9)	1(0.9)	1(0.9)
Wrists/hands	12(10.9)	11(10.0)	9(8.2)	5(4.5)	5(4.5)	1(0.9)
Lower back	30(27.3)	25(22.7)	25(22.7)	17(15.5)	14(12.7)	6(5.5)
Hips/thighs	5(4.5)	3(2.7)	3(2.7)	3(2.7)	1(0.9)	1(0.9)
Knees	6(5.5)	2(1.8)	3(2.7)	4(3.6)	3(2.7)	2(1.8)
Ankles/feet	11(10.0)	9(8.2)	8(7.3)	4(3.6)	3(2.7)	2(1.8)

### Problems preventing daily activity and seeking assistance

**Fig. 1:**
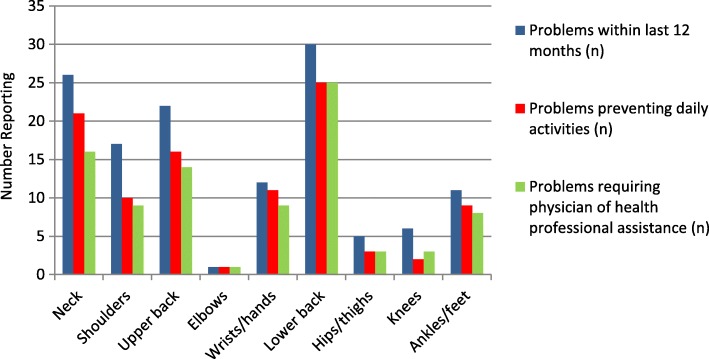
Regions of musculoskeletal problems (12 months) and proportion effecting daily activity and needs to seek professional assistance

The most common musculoskeletal problem preventing activities within the last 12 months were also the lower back (22.7%), neck (19.1%) and upper back (14.5%) in the total sample (*n* = 110; Table 2). This accounted for an average of 79% overall (between 73 and 83%) of those reporting a problem at these three sites (*n* = 62/78; Fig. 1). The wrists/hands (10.0%) were the 4th most common site preventing activities, followed by the shoulders (9.1%) and ankles/feet (8.2%).

**Fig. 2:**
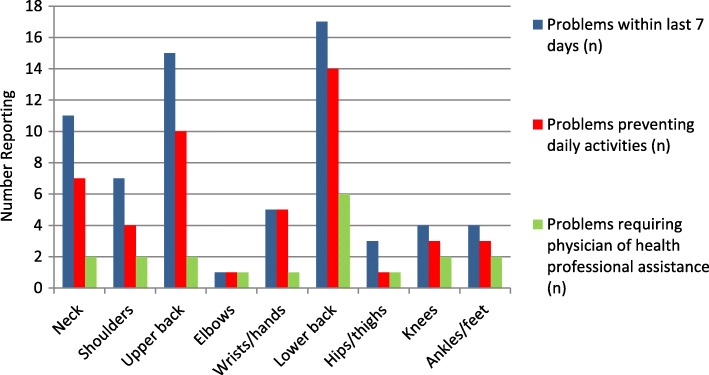
Regions of musculoskeletal problems (7 days) and proportion effecting daily activity and needs to seek professional assistance

Similarly, the most common musculoskeletal problems preventing activities within the last 7 days in the total sample also occurred in the lower back (12.7%), upper back (13.6%) and neck (6.4%), followed by the wrists/hands (4.5%), shoulders (3.6%), knees and ankles/feet (2.7%; Table 2). Lower back, upper back and neck problems preventing activities accounted for an average of 72% overall (between 64 and 82%) of those reporting a problem at these three sites in the immediate 7 day recall period (*n* = 31/43; Fig. 2).

The most common sites requiring assistance from a physician or health professional in the total sample (*n* = 110) in the last 12 months were the lower back (22.7%), neck (14.5%) and upper back (12.7%). This accounted for an average of 71% overall (between 62 and 83%) of those reporting a problem at these three sites (*n* = 55/78; Fig. 1). The shoulders (8.2%) and wrists/hands (8.2%) were the next more likely site requiring treatment. The lower back (5.5%) was the most common site reported to require treatment in the last 7 days, followed by the neck (1.8%), upper back (1.8%), shoulders (1.8%), knees (1.8%) and ankles/feet (1.8%; Table 2). Lower back, neck and upper back problems requiring treatment accounted for an average of 23% overall (between 5 to 35%) of those reporting a problem at these three sites in the immediate 7 day recall period (*n* = 10/43 Fig. 2).

While fewer students reported lower limb sites in the 12 month period, most students experiencing ankle/feet problems, reported the problems prevented daily activities (81.8%, *n* = 9/11) with almost three-quarters seeking physician or health professional assistance (72.7%, *n* = 8/11). Similarly, 75.0% of ankle/feet problems prevented daily activities (*n* = 3/4), and 50.0% sought physician or health professional assistance (*n* = 2/4), in the 7 day recall period.

### Factors associated with work-related musculoskeletal problems

Reported laboratory work-related musculoskeletal problems in 12 months were not associated with gender (*X*^*2*^(1) = 0.994, *p* = 0.319), year of study (*X*^*2*^(1) = 1.401, *p* = 0.492), and seated/ standing working positions (*X*^*2*^(1) = 4.319, *p* = 0.115). No differences were found between the year of study and hours of laboratory activity participation (F(2,107) = 1.731, *p* = 0.182), suggesting homogeneity in exposure hours. The association between self-reported poor posture and reported laboratory work-related musculoskeletal problems in 12 months (*X*^*2*^(1) = 22.491, *p* = 0.001) and 7 days (*X*^*2*^(1) = 10.553, *p* = 0.001) were significant. Students were also more likely to participate in ergonomic musculoskeletal exercises when experiencing a problem in the 12 month (*X*^2^(1) = 8.425, *p* = 0.004) and 7 day (*X*^2^(1) = 7.935, *p* = 0.005) recall periods.

### Student reported solutions

Thirty percent (*n* = 11/38) of students reporting a musculoskeletal problem, provided written comments at the end of the survey. Common themes about solutions to prevent laboratory work-related musculoskeletal problems were 1) better designed seating 2) fatigue management and 3) learning about better posture.

In the theme ‘better seating design’, students suggested *‘better seating should be made available in the laboratory’ (Participant 1), ‘the chairs are too high for the benches and result in students having to crouch to write neatly in their lab books’ (Participant 3)* and *‘I think that there should be better seats in the laboratory practicals besides stools as they do not support your back while sitting’ (Participant 3).*

Fatigue management theme comments included *‘reducing the amount of time spent in the laboratory….. and taking more breaks’ (Participant 8), ‘making the classes shorter’ (Participant 6)* and *‘I truly think that the laboratory practicals for medical science students should not be more than 2 hours’(Participant 9).*

Learning about better posture theme comments included *‘correcting posture with daily exercise’ (Participant 7)* and *‘education about ‘best’ postures in sitting up straight*’ *(Participant 11)* and *‘fixing your posture when in the laboratory’ (Participant 10)*. Seeking professional assistance was also considered part of the preventative strategy *‘people suffering from pain or discomfort need to see a doctor, and look for ways of preventing pain or further injury’ (Participant 5)*.

## Discussion

Work-related musculoskeletal injuries and disorders are a serious health and economic concern for individuals, the workforce and the community in general. Laboratory technicians are not immune, with 72 to 80% of laboratory work-related musculoskeletal problems reported in studies from Iran and India [16, 17] using the SNMQ [18].

This study investigating the prevalence, common sites and impact of work-related musculoskeletal problems amongst medical science students found concerning rates of self-reported musculoskeletal problems during their training. Results indicate that more than a third of medical science students experience work-related musculoskeletal problems early in their practical laboratory based training. While over a third of students reported one or more musculoskeletal problems occurring within the last 12 months, almost a quarter also reported a more recent musculoskeletal problem within the last 7 days. This prevalence is around half of that reported amongst full time laboratory technicians, despite students only working one-sixth of hours worked by full time workers [16, 17]. Furthermore, this is likely to be an underestimation of the workforce weekly hours with many workers reporting working in excess of 50 h a week [14]. While female gender is associated with high reported work-related musculoskeletal problems [14, 16], this study with medical science students was not consistent with this evidence.

### Musculoskeletal problems and consistency with workforce data

Consistent with previous research with laboratory technicians using the SNMQ, common sites for musculoskeletal problems in medical science students during laboratory activities, were the lower back (30% compared to 31 to 43%), neck (24% compared to 18 to 33%) and upper back (21% compared to 20%) [16, 17]. This is also similar to research from America, Ethiopia, Iran, Sweden and Switzerland indicating the neck (40–60%) and lower back (9–57%) were common sites musculoskeletal problems in laboratory technicians [14, 15, 19–22]. The high prevalence of back problems is also consistent with Australian data with back problems accounting for 33% of musculoskeletal injuries [1]. Workplace musculoskeletal problems occurring early in careers needs further investigation to understand the potential contribution to national economics costs, particularly given back injuries were estimated to cost $1.2 billion in Australia in 2008–2009 [25].

### Laboratory activities and prevalence of musculoskeletal problems

Repetitive and physical demands associated with laboratory activities are identified as contributing factors to the development of musculoskeletal problems [13]. Awkward and prolonged working postures with static loads on muscles have been implicated in the development of back, shoulder and neck problems [13], which is consistent with the common sites of reported problems by medical science students. Students who perceived they had poor postures were more likely to report musculoskeletal problems in both the 12 month and 7 day recall period. Students were also conscious about the ergonomic demands of laboratory tasks reporting the need for ‘*better seating design*’ and to ‘*learn about better posture’*.

Particular activities, such as pipetting have also been associated with musculoskeletal symptoms in hands, shoulders and the neck, with high usage and fatigue adding to the injury statistics [17, 22]. Medical science students also reported spending around a third of their time performing pipetting activities, which could contribute to their reported problems in the hands (12%), shoulders (15%) and neck (24%). Consistent with previous literature, poorly designed workstation height (for example, too high or low) used by laboratory technicians may have contributed to these musculoskeletal problems [17]. As the majority of medical science students used non- adjustable stools seating, the one size fits all model, may be exacerbating their musculoskeletal problems. Furthermore, medical science students voiced views about the need for better height adjustable chairs and back support, to avoid ‘*hunching’* during laboratory activities.

### Fatigue and workload related musculoskeletal problems

Fatigue and increased years within the profession has been indicated to be a contributor to workplace musculoskeletal problems [15, 22]. However this study found no difference in year of study or reported problems. The homogeneity of hours of laboratory activities across the years of study, which is a curriculum constraint, may offer one explanation for this difference. While the average hours of laboratory activities were only around six hours per week, this was sufficient to affect musculoskeletal heath. As such, fatigue management was considered a possible solution by students to take more breaks and limit class duration, suggesting an understanding of the accumulative effects of laboratory activities and the need for a proactive participatory culture [10, 13].

### Lower limb musculoskeletal problems

Lower limb musculoskeletal problems were less prevalent sites reported by medical science students however, the prevalence was similar to the literature. The prevalence of knees, ankles and foot problems in this study were between 5 and 10% compared to 10 to 20% in the workforce literature [15, 17, 22]. The association between lower limb problems and prolonged standing, and moving across the workplace for different equipment, such as fume cupboards and centrifuging equipment has been previously reported [15, 17]. In this study students reported 15% of their time in these lower limb loading tasks.

While some work-related musculoskeletal problems are less prevalent, they still can have impact on daily activities and need for medical assistance. For example, in the students reporting problems in the ankle and feet, almost all problems (82%) prevented them participating in daily activities and required them to seek medical assistance (73%). This raises concerns that undergraduate medical science students, already in their training, are experiencing work related musculoskeletal problems which impact on daily activities and require treatment regardless of the prevalence rate. In fact, across all sites of reported problems, the majority of problems prevented daily activity as well as requiring treatment in a 12 month and 7 day recall period.

### Musculoskeletal problems in early career professionals

Previous research findings have also shown that early career health professionals account for 45% of reported musculoskeletal injury, occurring within the first five years of practice [12]. It is concerning that the current study suggests medical science undergraduate students may be pre-exposed to musculoskeletal problems, heightening their risk of developing more severe musculoskeletal disorders early in their career. It is likely students in training are more vulnerable to work-related musculoskeletal problems, because of the lack of musculoskeletal conditioning, experience in the laboratory when learning new skills [12, 20] and stress to meet laboratory tasks and assessment schedules [13]. This leads to the importance of managing laboratory workplace ergonomics and training, even as part of university curriculums, particularly in light that musculoskeletal injury has been associated with burnout, workplace stress and health workforce shortages [4, 6, 7]. While this is the first study to investigate musculoskeletal problems experienced in medical science students during their university training, this study replicates the high prevalence of musculoskeletal workplace problems experienced in the laboratory professional workplace.

### Educational needs

To address the high prevalence of laboratory work related musculoskeletal problems, previous recommendations include the need for better ergonomic education within university curriculums and the workplace [14, 20]. While ergonomic improvements in equipment and workplace design may be needed, a participatory model approach [10, 20] is recommended at the job front. The intention to address postural issues and for individuals to learn how to manage their own postural variations, within different workstations they may be presented with. While only 10% of students provided written comments to address work-related musculoskeletal problems, it seems they are ready to engage in ergonomic education, with students indicating that *‘learning about better posture’* would help them manage musculoskeletal problems. Furthermore, this study found an association with reported problems and participation in ergonomic exercises. It is likely, that the many students seeking assistance for their musculoskeletal problems are being advised to undertake exercises to manage them. Further research is needed to investigate the acceptability, content and benefits of ergonomic postural education to prevent/manage musculoskeletal problems experienced during undergraduate medical science workplace training activities.

### Limitations

While this study was able to provide important data on reported musculoskeletal problems in medical science students, several limitations are noted. Only one class list from each year was used to send emails to students, without direct student contact to promote the research project. While the survey reached students across all years the response rate was low (38%). It is known students receive numerous emails through their student account and do not always prioritise these notifications. However, the response rate is relatively consistent with previous reported research (50%) investigating work-related musculoskeletal in the workforce [20]. It is possible that students with work-related musculoskeletal problems were more likely to respond, however 66% of respondents who completed the survey did not report any laboratory work-related musculoskeletal problem.

As in any cross-sectional study, causal relationships cannot be determined. Similarity in hours of laboratory participation across the years of the study was a curriculum constraint and also limited exposure investigation. While we were able to investigate associations between musculoskeletal problems and key variables, causation could not be established, nor were ergonomic audits of workstations and activities undertaken. Furthermore, even though the response rate was comparable to other studies, the results need to be considered with some caution given the response rate may not be fully representative of the total student cohort.

While students provided rich information about their experience within the open ended questions, this only accounted for 10% of the total sample and 30% of students reporting problems, thus may not comprehensively represent the opinion of the whole sample. Further studies monitoring musculoskeletal problems over time with more complete response rates are needed. As students were studying in one program at one university, it is possible that results may differ in different programs and contexts.

## Conclusions

University medical science students in our study, already experience symptoms of work-related musculoskeletal problems, during their laboratory training activities. The lower back, neck and upper back have the highest prevalence. While problems may be considered part of the process of learning new tasks, students are reporting laboratory work-related musculoskeletal problem that are impacting their daily activities. While the response rate was low and thus may not fully represent the sample the impact of reported musculoskeletal problems is concerning. The majority of students reporting problems indicated the cessation of daily activities with many seeking medical or health professional assistance. Sustained awkward and constrained working postures during laboratory practicals have been implicated, with students suggesting need for better seating and education about safer work postures.

Taking corrective measures to address posture and working positions may reduce the prevalence of musculoskeletal problems. Further research is needed to investigate the stages of musculoskeletal problems experienced by students, and strategies which analyse the effects of exercise on working performance and the ergonomic impact of student’s workstations. Identifying and addressing the extent of musculoskeletal workplace problems is essential to facilitate sustainable healthy and productive workforces.

## Abbreviations

IQR: Interquartile Range
n: Number of Participants
SD: Standard Deviation
SNMQ: Standardised Nordic Musculoskeletal Questionnaire
